# Clinics Given at the Tri-State Dental Meeting

**Published:** 1901-10-15

**Authors:** 


### CLINICS GIVEN AT THE TRI-STATE DENTAL MEETING.

### NEW FORMS OF SCALERS.

BY DR. C. R. BUTLER, CLEVELAND, O.

These new instruments cover a wide field of usefulness in the treatment of of pyorrhea alveolaris. They are especially adapted to the many extreme cases, as from the length and size of the blades all surplus metal is taken away from the line of vision. The instruments also facilitate the work that has to be done through the sense of touch. They were all designed and constructed by the doctor himself.

### FILLING TEETH WITH IMPROVED TIN FOIL.

BY DR. H. L. AMBLER, CLEVELAND, O.

Dr. Ambler showed that with his own make of cohesive tin foil, a filling in any cavity in any tooth could be made one solid mass from base to surface, so that if the filling was removed, by breaking the tooth, it could be put on an anvil and forged out flat without breaking in pieces. After partly filling a cavity he burnished the surface, then built on more tin so firmly that it could not be pried off. This foil is more tenacious, cohesive, and makes a harder filling than any similar foil. It is used entirely for technic work in a few dental colleges in place of cohesive gold, because it teaches the use of tin and cohesive gold at the same time, as the tin is manipulated with the same instruments and hand mallet as used for cohesive gold.

### PORCELAIN CROWNS.

BY DR. C. E. REDMOND, PERU, IND.

In making this crown no band in used, but the root is concaved mesio-distally. A platinum disk is then burnished over the face of the prepared root and a pin is soldered to the platinum disk. A porcelain facing is secured and soldered to the platinum disk. Fill in the back with Head's porcelain body, and shape and fuse. A round file is used in the engine to shape the root, and one need not remove the enamel near the gum when making this style of crown.

### CARVED CUSP FOR GOLD CROWN.

BY DR. J. E. NYMAN, CHICAGO, ILLS.

A practical method of making solid cusps for shell crowns consists of moulding the cusp in modelling compound to conform to the opposing tooth, then securing a mould in moldine and casting a cusp of scrap gold.

### ANTISEPTIC FLOSS TOOTHPICK.

BY DR. J. W. COWAN, GENESEO, N. Y.

This floss toothpick is designed to remove particles of food from the spaces between the teeth, and to clean and sterilize those surfaces which the toothbrush can not reach. The appliance consists of a hollow metallic case about the size and shape of a small penknife, the the upper and lower parts being hinged together. A five-yard ball of floss silk, saturated with an antiseptic, is sealed in small glass vials. Each vial when in use is held removably in place by a small spring clip within the butt end of the case, and a spring wire frame, adapted to hold a section of floss securely between its arms, mounted pivotally in the forward end of the case. When it is desired to operate the device, the case is opened, the spring frame turned outward, resting in an opening in the end of the casing, and the cover closed, thus holding the frame in operative position. Successive lengths of the silk as desired are drawn through a slit in the cork which seals the vial, and out through the same opening, and wrapped two or three times into the little hooks on either end of the spring frame, thus providing a taut section of silk which may be easily forced between any of the teeth. When the silk has been exhausted the empty vial is slipped out of its position, thrown away and a fresh one substituted. When not in use the case is opened, the spring frame folded inward and then closed again, thus protecting the silk and frame from dust, etc., while being carried in the pocket.

### CORRECTION OF DENTO-FACIAL DEFORMITIES.

BY CALVIN S. CASE, M.D., D.D.S.

Synoptic report of a lecture before Tri-State Dental Meeting, Indianapolis, June, 1901.

Dr. Case illustrated his lecture by showing with the lantern pictures of three cases in which he had not only corrected the occlusion of the teeth, but had also very materially modified the abnormal facial lines to conform to the idealistic. He also showed two cases where the treatment was very materially influenced by skiagraph pictures which located hidden, unerupted teeth and made it possible to bring them into the arch.

He also exhibited pictures illustrating a case of cleft palate in boy of sixteen years, in whose mouth the occlusion was very unsatisfactory. There was great protrusion of the upper, and retrusion of the lower teeth, producing a great prominence of the nose and upper lip. By judicious extraction of some upper defective teeth a very satisfactory profile was secured.

Another was a case of great protrusion of the incisor and spreading of the arch in a patient thirty-five years of age, who was badly affected with pyorrhea alveolaris. In this case, by extraction and retraction, and the insertion of a bridge, a very satisfactory result was secured.

Prof. Brophy, of the Chicago Dental College, gave a very interesting lecture on his method of treating cleft palate cases. The lecture was illustrated by lantern slides and was very effectively presented. It was the same lecture which he presented at the Paris International Dental Congress, a synopsis of which appeared in the May REGISTER. It was printed in full with illustration in the April *Dental Cosmos*. The method is full of interest to every dentist, as it demonstrates the possibility of the certain removal of this dreadful misfortune from those afflicted, with a minimum of adverse results.

Because of the fact that the rooms had to be darkened for the illumination of the pictures it was impracticable to make any considerable report of these lectures.

